# Self-Remedied

**Published:** 1875-07

**Authors:** 


### SELF-REMEDIED.

In the May number of this Journal we mentioned two of the most common causes of diseases peculiar to females. We affirmed that in many cases, and it may be no exaggeration to say that, in the majority of instances, the patients are themselves the authors of the ills from which they suffer. It is now our purpose to suggest some of the means by which these ills may remedied, or in a great degree alleviated. Our remarks will be limited to such measures as may be adopted without the aid of a physician; since we hope to show that the disease which has been *self-inflicted* may, to a great extent, be *self-remedied.* Of course the *corset* must be dispensed with. We cannot expect to remedy any evil while we hold on to the cause thereof. We are aware that the corset is regarded as an essential part of a lady's apparel. It is "fashionable," and that settles the question. If a woman choses to violate the laws of health and life for the sake of being in the fashion, she may expect to suffer the penalties, and she ought never to complain about her poor health. But aside from the looks, and independent of the fashion, many think they can not do without the support which the corset affords. If they leave it off they feel as if they "would fall to pieces." This very unpleasant feeling results from one of the injurious effects of the corset. The constant pressure exerted upon the muscles of the chest and body produces a condition of partial paralysis of those muscles. It is, in part, their function to support the internal organs, and give symmetry and form to the body. They perform this office perfectly in man, and would in woman if they were not interfered with and deprived of their vigor by the tightly laced corset.

Any muscle, or set of muscles, lose their tone and, in time, waste away if unused. Let an arm be placed across the chest and confined there by bandages, or in a sling for a few weeks only, and then released and allowed to take its natural position, and what would be the sensation? It would feel as if it was about to drop off. It would hardly have strength to sustain its own weight. In fact, the muscles of the arm would be in the same enfeebled condition as those of the chest from the wearing of corsets.

Now, this very disagreeable fall-to-pieces feeling will soon pass away. The muscles will regain their strength and resume their function of supporting the body very soon after they have a chance to do so. Bathing the chest and back with water, and friction with the hand, will aid materially the course of restoration and invigoration.

It is important that the pelvic organs be relieved from the weight of the under clothing. These should be entirely supported by the shoulders. The constant downward pressure of skirts is a common cause of internal displacements.

Sea bathing is often of great benefit in these diseases. But sea bathing is not always practicable. The season of the year, and distance of the patient from the ocean wlll prevent the great majority of those afflicted from resorting to this very effectual remedy. And then, so few persons will exercise any discretion as to the time they should remain in the water, and as to its temperature, that we often refrain from advising a measure that may prove hurtful from the lack of proper care in its use. But, thanks to the art of the chemist, it has lately become a very easy matter to get the advantages of sea-bathing, without its perils, in our own homes. We recommend, with great confidence to ladies suffering from the disease under consideration the use of the *sea-salt* prepared by the well known chemist and pharmacist, Mr. A. J. Ditman, of this city. This salt is prepared from sea-water, is free from all impurities, and when dissolved in the proper quantity of water affords a delightful and invigorating bath. The sitz bath is appropriate in all cases of "weakness." The water should be of a temperature most agreeable to the patient. Simple sponging of the back and about the hips and around the fore part of the person will prove very beneficial. These baths, in whatever form taken, full bath, sitz, or sponge, may be used daily, either on retiring or on arising from bed, as most convenient. We have much faith in the efficacy of bathing, and especially in the use of the hip bath, or the sitz bath, as it is usually termed. By its judicious use the muscles of the lower part of the body are strengthened, congestion and inflammation of internal organs relieved and the troublesome and debilitating discharges, so often present, are checked. But for the latter we cannot too strongly recommend the use of injections (vaginal) of pure water, and sometimes of weak solutions of alum, or other astringents. The only efficient instrument for this purpose is the long rubber tube syringe. A pint or quart should be used at a time, and repeated daily.

We cannot too earnestly urge the importance of care and rest during the times of the monthly indisposition Many women would escape a host of ills if they would take proper care of themselves at these times. And by proper care, we mean simply *rest*, and the avoiding of work and all fatiguing exercise. We endorse fully the sentiment of an intelligent Irish woman that "it is against nature for a woman to work hard when she is unwell." It surely is "against nature;" and but few can sin in this way, not to mention other practices that are *crimes* against nature, without sooner or later suffering the penalties.

This necessary care and repose may involve the neglect, or postponement, of certain important household duties. It may "put back the work" a day or two; it may interfere with some projected excursion or pleasure party, but the advantages to be gained in the way of health and strength ought to be duly considered. It seems that some women have no idea of the comparative importance of things. The most trivial affairs are, in their estimation, "enterprises of great pith and moment." Each domestic undertaking must be prosecuted at all hazards. Because it is Friday and Friday is "sweeping day"

the woman, whose condition requires that she should rest quietly on the sofa, will faithfully observe the day by wielding the broom and dusting brush from cellar to attic. The risks to her own health do not receive a moments consideration when she thinks how "perfectly awful every thing will look" if the sweeping is not done. An imprudence of this kind may cause a life of suffering. Mothers should carefully guard their daughters against unusual exercise or excitement of any kind at these periods. Just here we wish to mention that one of our greatest modern household conveniences is chargeable with ruining the health of many young girls. Of course we mean the *sewing machine.* There is hardly any work more injurious to a woman at the times of her indisposition than running a machine.

The foregoing remarks are not intended merely for those who suffer from "irregularities." They are also directed to the perfectly healthy. These cannot act "against nature" and retain their health. Women should know that the uterus is remarkably prone to get into bad habits. Certan phenomena of disease are said to recur from habit. In this case a hemorrhage may be caused by some imprudence during the monthly period. The same may very likely be repeated on the next occasion, and the next, and that without a repetition of the cause. A morbid habit has been formed, which, like all bad habits, is not so easily corrected. We might name other examples of disordered action becoming habitual. This will suffice to impress upon those of our lady readers who are well, the importance of guarding carefully that most precious blessing—health.

It is not our intention to prescribe any of the few medicines which we have found useful in the common diseases of females. We cannot do so without entering into the special features of individual cases; and besides we would have our lady readers learn that they can do a great deal for themselves without the help of drugs. And we would urge most seriously the avoiding of *instrumental* treatment. This should be the final resort, and only in extreme cases and then in the hands of an experienced and conscientious physician. There are many practitioners, in city an[illegible] country, who make a specialty [illegible] female diseases. They indulge in a great deal of *heroic* practice, operations, &c. An unprincipled man has an opportunity of deceiving his patients more in this specialty than in any other branch of the profession. He easily enumerates the symptoms which nearly all women experience more or less, and some of which are sure to exist in any patient. He then urges the necessity of "an examination," and that examination is certain to reveal a condition that calls for immediate treatment. He thereby gains a patient; and the unfortunate woman, who was, until then, in the enjoyment of a tolerable degree of health, finds herself down sick from the time she began to be doctored. It should be remembered that considerable deviation from the natural state of the uterus may exist and still the general health not be seriously deranged. For instance, the organ may be enlarged or partially displaced, but if no serious inconvenience attends, it is much better to let things alone than to go to the doctors.

If women who are troubled with symtoms which indicate some form of female disorder, would take the care of themselves which common sense indicates, and use persevereingly the remedies suggested above, we believe that nine tenths of them, and perhaps a greater proportion, would be better off than if subjected to the treatment of the most scientific, or the most empiracle, physician.

